# *Retama monosperma n*-hexane extract induces cell cycle arrest and extrinsic pathway-dependent apoptosis in Jurkat cells

**DOI:** 10.1186/1472-6882-14-38

**Published:** 2014-01-24

**Authors:** Lamiae Belayachi, Clara Aceves-Luquero, Nawel Merghoub, Youssef Bakri, Silvia Fernández de Mattos, Saaïd Amzazi, Priam Villalonga

**Affiliations:** 1 Cancer Cell Biology Group, Institut Universitari d’Investigació en Ciències de la Salut (IUNICS), Edifici Cientificotècnic, Ctra Km 7,5, Valldemossa, Illes Balears, Spain; 2 Departament de Biologia Fonamental, Institut Universitari d’Investigació en Ciències de la Salut (IUNICS), Universitat de les Illes Balears, Edifici Cientificotècnic, Ctra Km 7,5, Valldemossa, Illes Balears, Spain; 3 Biochemistry Immunology Laboratory, Faculty of Sciences, Mohammed V-Agdal University, Rabat, Morocco

**Keywords:** *Retama monosperma*, Acute T-cell leukemia, Cytotoxicity, Apoptosis, Bioactive compounds

## Abstract

****Background**:**

*Retama monosperma* L. (Boiss.) or *Genista monosperma* L. (Lam.), locally named as “R’tam”, is an annual and spontaneous plant belonging to the Fabaceae family. In Morocco, Retama genus is located in desert regions and across the Middle Atlas and it has been widely used in traditional medicine in many countries. In this study, we show that *Retama monosperma* hexane extract presents significant anti-leukemic effects against human Jurkat cells.

**Methods:**

Human Jurkat cells, together with other cell lines were screened with different concentrations of *Retama monosperma* hexane extract at different time intervals. Growth inhibition was determined using luminescent-based viability assays. Cell cycle arrest and apoptosis were measured by flow cytometry analysis. Combined caspase 3 and 7 activities were measured using luminometric caspase assays and immunoblots were performed to analyze expression of relevant pro- and anti-apoptotic proteins. GC-MS were used to determine the chemical constituents of the active extract.

**Results:**

*Retama monosperma* hexane extract (Rm-HE) showed significant cytotoxicity against Jurkat cells, whereas it proved to be essentially ineffective against both normal mouse fibroblasts (NIH3T3) and normal lymphocytes (TK-6). Cytometric analysis indicated that Rm-HE promoted cell cycle arrest and apoptosis induction accompanied by DNA damage induction indicated by an increase in p-H2A.X levels. Rm-HE induced apoptosis was partially JNK-dependent and characterized by an increase in Fas-L levels together with activation of caspases 8, 3, 7 and 9, whereas neither the pro-apoptotic nor anti-apoptotic mitochondrial membrane proteins analyzed were significantly altered. Chemical identification analysis indicated that α-linolenic acid, campesterol, stigmasterol and sitosterol were the major bioactive components within the extract.

**Conclusions:**

Our data suggest that bioactive compounds present in Rm-HE show significant anti leukemic activity inducing cell cycle arrest and cell death that operates, at least partially, through the extrinsic apoptosis pathway.

## Background

Medicinal plants used in traditional or folk medicine and their active compounds, used in conventional medicine, constitute the basis for the treatment of many diseases including cancer, one of the major causes of mortality throughout the world. Statistics have estimated that cancer will cause 83.2 million deaths between 2005 and 2015 [1]. Clinical applications have, for many decades, demonstrated their therapeutic effects. In fact, 40% of all available anticancer drugs are natural products or natural product-derived mimics, such as paclitaxel (from *Taxus brevifolia*), camptothecin (from *Camptotheca acuminata*), podophyllotoxin (from *Podophyllum emody*) and vinblastine (from *Catharanthus roseus*), which are widely-used anticancer agents [2].

*Retama monosperma* L. (Boiss.) or *Genista monosperma* L. (Lam.), locally named as “R’tam”, is an annual and spontaneous plant belonging to the Fabaceae family. The genus Retama includes four species with a geographic distribution in the Mediterranean area, North Africa, and the Canary Islands [3]. In Morocco, Retama genus is largely located in desert regions and the Middle Atlas [4]. This plant is used in traditional medicine in many countries, as purgative, vermifuge, antihelmintic, and abortive [5]. Moreover, several studies have investigated *Retama* genus for various pharmacological effects, including hypoglycemic and diuretic [4,6], cytotoxic [7,8], antioxidant, antiviral [9], antihypertensive [10], anti-inflammatory [11] and antitumor activities [12].

T-cell malignancies are highly aggressive neoplastic disorders that are generally resistant to conventional chemotherapy with a high rate of relapse and currently no efficient targeted therapies available for these diseases [13]. In order to extend the treatment options and ultimately improve survival for patients with leukemia, it is imperative to increase the therapeutic arsenal of targeted therapies involving apoptotic cell death, that has been proposed as an efficient mechanism by which malignant tumor cells can be removed upon treatment with chemotherapeutic drugs without accompanying a local damaging inflammatory response [14].

In chemotherapeutic drug-induced apoptosis of tumor cells, three different death signaling pathways can lead to apoptosis: the extrinsic death receptor-dependent pathway [15], the intrinsic mitochondria-based pathway [16], and the intrinsic endoplasmic reticulum (ER) stress-mediated pathway [17].

In this study, we show that *Retama monosperma* hexane extract presents selectively an anti-leukemic effect, as indicated by its dramatic effects on Jurkat, but not other human cancer cells of various origins. We describe herein the major cellular effects of Rm-HE leading to cell cycle arrest and extrinsic pathway-dependent apoptosis. Finally, we suggest potential bioactive compounds responsible for these effects upon the determination of the chemical components of the extract by GC/MS.

## Methods

### Plant material

Leaves of *Retama monosperma L.* (Boiss.) were collected in March 2009 from Sidi Boughaba reserve in Mehdia-Rabat (Morocco). The plant was identified at the Scientific Institute of Rabat by Prof. M. Fennane, and the specimen was deposited in the Scientific Institute herbarium under the voucher specimen reference N° RAB78140.

### Preparation of hexanic extract

The powder of the dried plant was extracted successively using a Soxhlet apparatus with *n-*hexane to obtain hexanic extract (Rm-HE). The resulting extract was then evaporated by a Rotavapor to obtain the dried extract and was kept at -20°C until use.

### Cell culture

Jurkat, TK6 and Jeko-1 cells were maintained in RPMI-1640 with L-Glutamine and HEPES (Biological Industries Ltd., Kibbutz Beit Haemek, Israel). LN229, T98G, U87MG, SW620, SW480, U2OS, PC-3 and NIH3T3 cells were maintained in DMEM High Glucose (4.5 g/l) with L-glutamine (Invitrogen, Carlsbad, CA). All cells were grown in a humidified incubator at 37°C with 5% CO2. RPMI and DMEM were supplemented with 10% heat inactivated foetal bovine serum and 100 units/ml penicillin/streptomycin (Biological Industries Ltd., Kibbutz Beit Haemek, Israel). All cell lines were subconfluently grown and passaged, routinely tested for mycoplasma contamination and subjected to frequent morphological tests and growth curve analysis as quality-control assessments. All cell lines were treated at a prophylactic concentration of 5 μg/ml with Plasmocin™ (Invivo Gen, San Diego, CA).

### Drugs and inhibitors

Doxorubicin (Developmental Therapeutics Program, Division of Cancer Treatment and Diagnosis, National Cancer Institute, Bethesda, MA), Q-v-Ophand and SP600125 (Calbiochem, Darmstadt, Germany) were added directly to the media at the indicated concentration and cells were harvested or analyzed at the time points indicated in the figure legends.

### Cell viability assays

The number of viable cells in culture was determined based on quantification of ATP, which signals the presence of metabolically-active cells, using the Cell Titer-Glo luminiscent assay kit (Promega, Madison, WI, USA), which is quicker than other commonly-used methods to measure the number of viable cells (such as MTT, alamar Blue® or Calcein-AM) that require prolonged incubation steps to enable the cells’ metabolic machinery to convert indicator molecules into a detectable signal. Following the manufacturer’s instructions, the cells were plated in 96-well plates, treated 24 h later with extracts dissolved in DMSO for the indicated times and concentrations followed by addition of Cell Titer-Glo reagent. Luminiscence was detected using a multi-well Synergy Mx scanning spectrophotometer (Biotek, Winooski, VT).

### Cell cycle analysis

Cell cycle analysis was performed using propidium iodide staining. Briefly, cells were washed in phosphate-buffered saline (PBS) and fixed in 70% ethanol. Fixed cells were then washed twice in PBS and stained in propidium iodide (50 μg/ml) in the presence of 50 μg/ml RNase A (Sigma-Aldrich, St Louis, MO, USA), then analyzed by flow cytometry using a FACScan (Coulter Epics XL-MSL; Beckman Coulter, Fullerton, CA, USA) and winMDI software.

### Annexin V-FITC/propidium iodide flow cytometric analysis

Analysis of phosphatidylserine externalization in apoptotic cells was determined by an Apo Target Annexin-V-FITC Apoptosis kit (Invitrogen, Carlsbad, CA), according to the manufacturer’s instructions. 2 × 10cells were seeded in 6-well plates and treated with 50 μg/ml of Rm-HE for 48 h. They were then collected and suspended in 100 μl of Annexin V-binding buffer. 5 μL of Annexin-V-FITC and 10 μL of propidium iodide were added and incubated 15 min at room temperature in the dark. Flow cytometry analysis was carried out using a FACScan (Coulter Epics XL-MSL; Beckman Coulter, Fullerton, CA, USA) and winMDI software.

### Caspase activity analysis

Enzymatic activity of caspases was determined by measurement of caspases-3 and 7 activity by means of the luminometric Caspase-Glo 3/7assay (Promega, Madison, WI, USA) according to the manufacturer’s protocol using a Synergy HT multi-detection microplate reader (Bio-Tek, Winooski, VT, USA).

### Gel electrophoresis and immunoblotting

Cells were harvested in a buffer containing 50 mMTris-HCl pH 7.4, 150 mMNaCl, 1 mM EDTA and 1% (v/v) Triton X-100 plus protease and phosphatase inhibitors. Protein content was measured by the Bradford procedure. Cell lysates were electrophoresed in SDS-polyacrylamide gels. After electrophoresis the proteins were transferred to Immobilon-P strips (Millipore, Billerica, MA) for 2 h at 60 V. The sheets were pre-incubated in TBS (20 mMTris-HCl pH 7.5, 150 mMNaCl), 0.05% Tween 20 and 5% defatted milk powder for 1 h at room temperature and then incubated for 1 h at room temperature in TBS, 0.05% Tween 20, 1% BSA and 0.5% defatted milk powder containing the appropriate antibodies: pH2A.X (#9718, 1:1000), Cleaved Caspase-3 (#9464, 1:1000), Cleaved Caspase-7 (#8438, 1:1000), Cleaved Caspase-9 (#7237, 1:1000), Caspase-8 (#9746, 1:1000), Bcl-2 (#2870, 1:1000), Bcl-xl (#2764, 1:1000) and Mcl-1 (#5453, 1:1000) from Cell Signaling (Beverly, MA); Bax (BD610391, 1:1000) and Bad (BD610982, 1:1000) from BD Transduction Laboratories™ (California, USA); Bim (22–40, Millipore, Billerica, MA, 1:1000); Fas-L (sc-6237, 1:1000), JNK (sc-137018, 1:1000) and _p_JNK (sc-135642, 1:1000) from Santa Cruz (Santa Cruz, CA) and β-tubulin (T0198, Sigma-Aldrich, St. Louis, MO, 1:4000). After washing in TBS, 0.05% Tween 20, the sheets were incubated with a peroxidase-coupled secondary antibody (Dako, Glostrup, Denmark, 1/2000 dilution,) for 1 h at room temperature. After incubation, the sheets were washed twice in TBS, 0.05% Tween 20 and once in TBS. The peroxidase reaction was visualized by the enhanced chemiluminiscence detection system (Millipore, Billerica, MA).

### Derivatization for GC/MS analysis

For this purpose 100 μl of the extract was dried with N_2_ gas, then 100 μl of derivatization agent (N, O-bis (trimethylsilyl) trifluoroacetamide with 1% of trimethylchlorosilane) was added, mixed and heated 10 minutes at 60°C.

### Gas chromatography/mass spectrometry (GC/MS) analysis

The GC-MS analyses of *Retama monosperma* hexanic extract (Rm-HE) were carried out at the Instrumental Technical Services of the “Estación Experimental del Zaidín” (CSIC, Granada, Spain). Briefly, 1 μl of the derivative solution was injected in a Varian 450GC coupled to 240 Ion Trap Mass Spectrometer as detector. The injection conditions were: splitless mode with 1 minute duration pulse, the injector temperature was 250°C; the He column flow was 1 ml/minute in a capillary column (Varian Factor Four VF-5 ms 30 m×0.25 mm×0.25 μm). For Mass spectrometry conditions, the EI ionization was 70 eV, the transfer line was at 280°C and the Trap at 240°C, mass range acquisition was from m/z 50 to m/z 500 and cared in Full Scan mode. Qualitative analysis of compounds was based on the comparison of their spectral mass and their relative Retention time with those of NIST08 mass spectra database and Kovats RI on the chromatograms recorded in Full Scan or in SIM mode using the characteristics ions. Quantitative analysis was realized by integration of peaks and calculated as percent of total identified area on the TIC chromatograms.

### Statistical analysis

Data are presented as means ± SD of at least three different assays performed in triplicate. IC_50_ value and the statistical significance of differences by Student’s *t* test were assessed using GraphPad Prism (GraphPad Software Inc. La Jolla, CA). Statistically significant differences are indicated by ***P, 0.001, **P, 0.01 and *P, 0.05, respectively.

## Results

### Analysis of the cytotoxic activity of Rm-HE against human cancer cell lines

**Figure 1:**
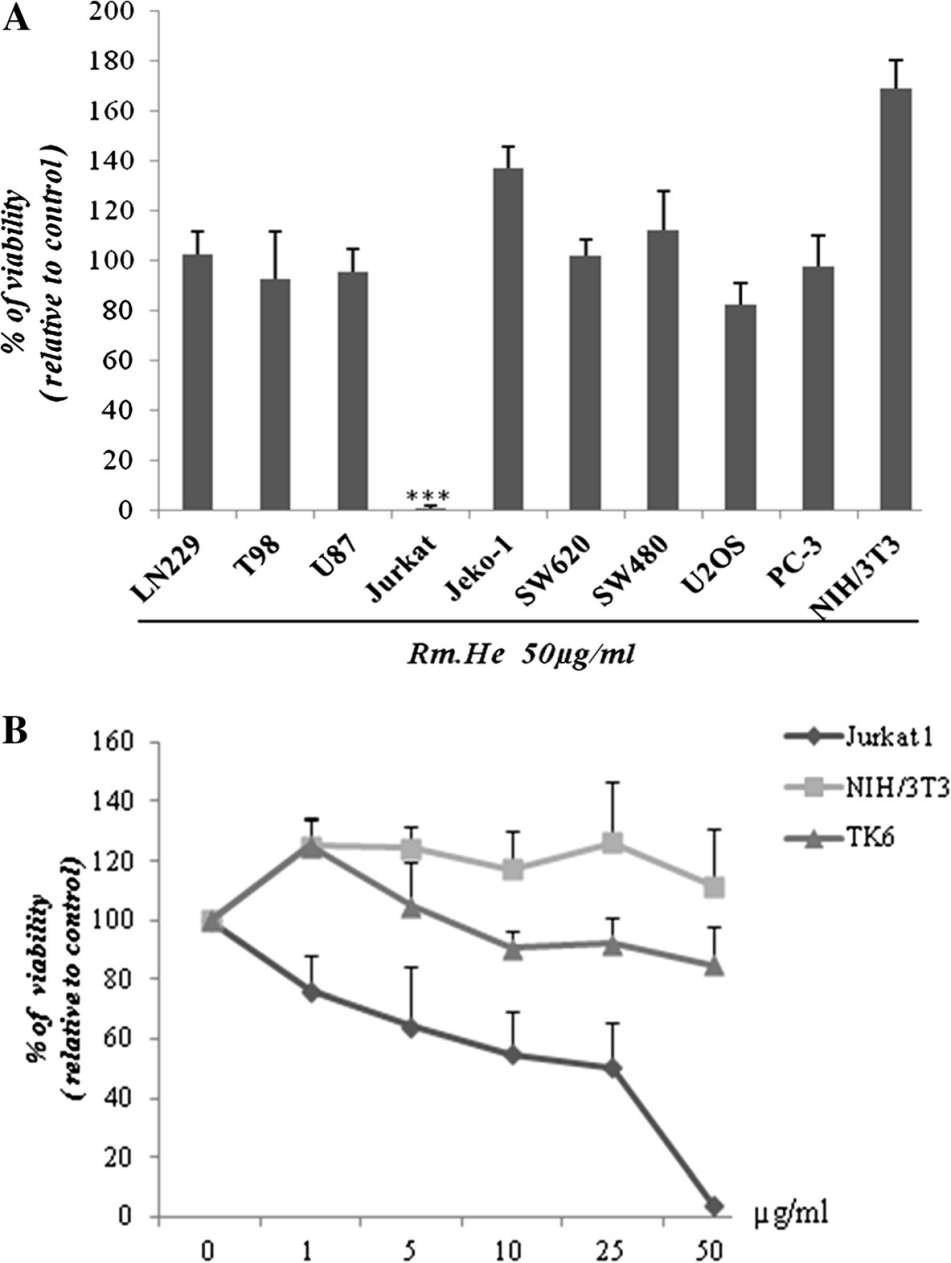
**Analysis of the cytotoxic activity of Rm-HE against human cancer cell lines. (A)** Cytotoxic activity of *Retama monosperma* Hexanic Extract (Rm-HE) in a panel of cancer and non-transformed cell lines. LN229, T98, U87, Jurkat, Jeko-1, SW620, SW480, U2OS, PC-3 and NIH3T3 cells were incubated for 48 h with 50 μg/ml of (Rm-HE); Results represent the mean ± SD of at least 3 independent experiments indicating the percentage of viable cells relative to vehicle-treated (control) cells. **(B)** Dose-response analysis of *Retama monosperma* Hexanic Extract (Rm-HE) and IC_50_ determination on Jurkat cells. Cells were treated with Rm-HE for 48 h at different concentrations ranging from 1 to 50 μg/ml; IC_50_ value (μg/ml) was determined graphically from the viability curves, NIH3T3 and TK6 cells were used as control. Data are expressed as means ± SD of triplicate determinations.

To investigate the potential effect of Rm-HE extract against cancer cells, various human cancer cell lines of different origin were screened to assess the cytotoxic activity of Rm-HE. Non-tumoral cell lines NIH3T3 and TK6 were also tested as control. Interestingly, Rm-HE extract was dramatically effective against Jurkat cells whereas it induced only modest or negligible effects in the other tested cell lines (Figure 1A). We next performed a dose-response (0-50 μg/ml) viability assay in Jurkat cells in order to calculate the IC_50_ for this cell line, using NIH3T3 and TK6 cells as controls. The obtained cell growth curves in Figure 1B prove that Rm-HE exerts a specific dose-dependent inhibitory effect on cell proliferation in Jurkat cells (IC_50_ = 34.44 ± 3.88 μg/ml). In agreement with our previous results, the extract exhibited no effects on NIH3T3 and TK6 (non-transformed cells lines) and a dose of 40 μg/ml was chosen for further mechanistic studies in Jurkat cells.

### Analysis of cell cycle effects of Rm-HE in Jurkat cells

**Figure 2:**
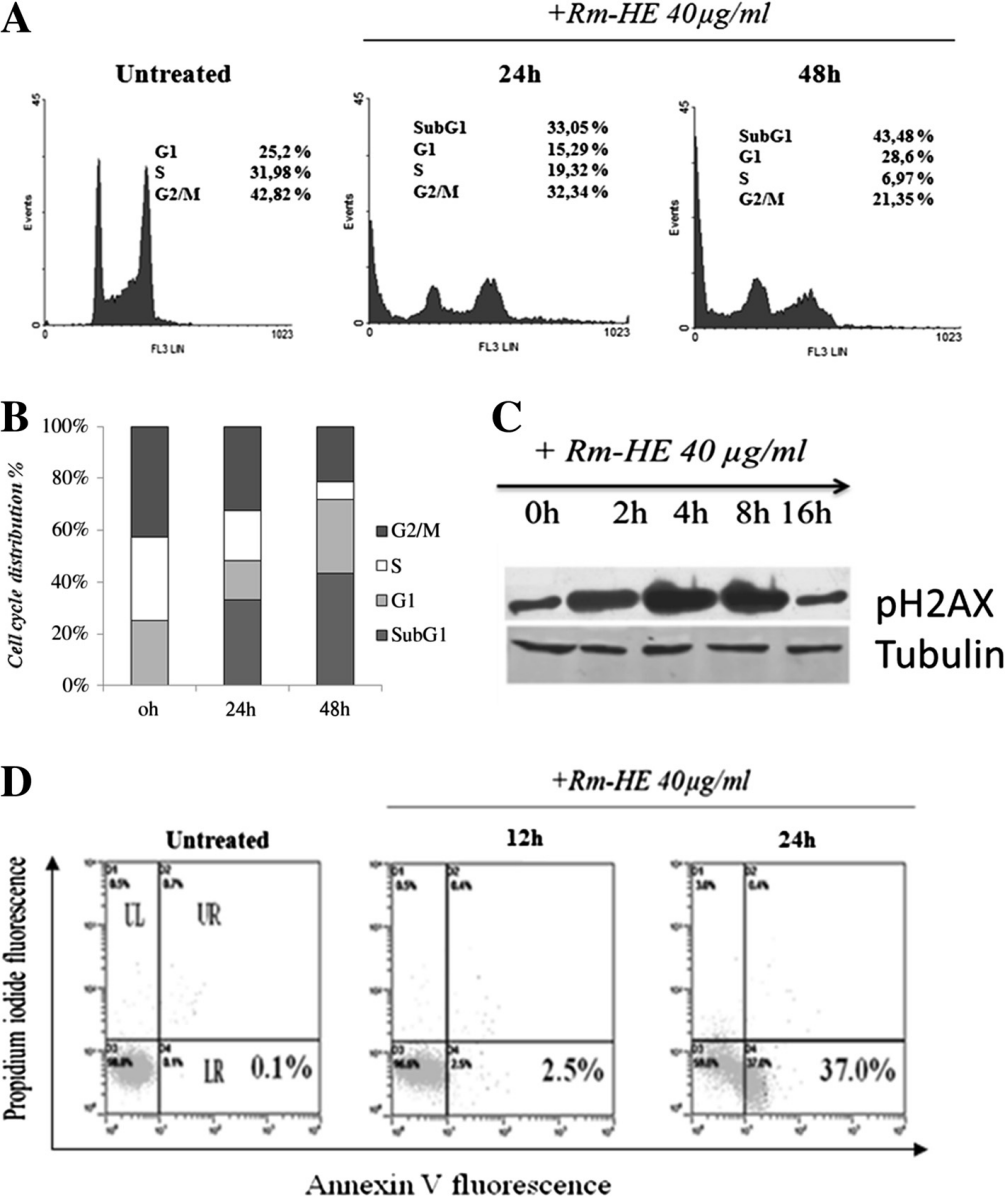
**Analysis of cell cycle effects and cell apoptosis of Rm-HE in Jurkat cells. (A)** Cell cycle analysis of Jurkat cells treated with *Retama monosperma* Hexanic Extract (Rm-HE) by flow cytometry. Jurkat cells (4 × 10 cells/ml) were incubated with 40 μg/ml of Rm-HE as indicated for 24 h and 48 h. Cells were harvested and their DNA content analysed by flow cytometry as described in Materials and Methods. The cell cycle distribution is shown for each experimental condition. **(B)** The graph summarizes the percentage of each phase in control, 24 h and 48 h-treated cells, respectively. **(C)** Effect of Rm-HE on pH2A.X levels. Cells (4 × 10cells /ml) were treated with 40 μg/ml Rm-HE for 0, 2, 4, 8 and 16 h. p-H2A.X levels in cellular extracts were detected by immunoblot with specific antibodies. Tubulin was used as an internal control. **(D)** Effect of Rm-HE in Jurkat cell apoptosis analyzed by flow cytometry. Cells were treated with 40 μg/ml of Rm-HE for 12 h and 24 h. The x-axis shows Annexin V-FITC staining and y-axis indicates Propidium iodide staining; Lower left (LL) quadrant: viable cells; lower right (LR) quadrant: early apoptotic cells; upper left (UL) quadrant: necrotic cells, upper right (UR) quadrant: late apoptotic cells.

In order to investigate how Rm-HE affects cell cycle distribution, Jurkat cells were treated with a concentration of 40 μg/ml for 24 and 48 h. As shown in Figures 2A and B, Rm-HE effectively reduced the proportion of S-phase cells while strongly increasing the proportion of sub-G1 cells (33.05% at 24 h and 43.48% at 48 h). To elucidate the possible mechanism of Rm-HE induced sub-G1 population, we analyzed the presence of DNA damage by monitoring p-H2A.X levels. As shown in Figure 2C, increased levels of p-H2A.X were detected in Jurkat cells after only 4 h, suggesting that Rm-HE treatment induced DNA damage. Double Annexin V/Propidium Iodide staining was next performed in order to analyze and quantify cellular death. Upon exposure to Rm-HE, a time-dependent increase in the number of Annexin V-positive cells (37%) at 24 h was observed (Figure 2D). Taken together, these data indicate that Rm-HE induces DNA damage accompanied by cell cycle arrest and apoptosis in Jurkat cells.

### Effects of Rm-HE on apoptosis induction in Jurkat cells

**Figure 3:**
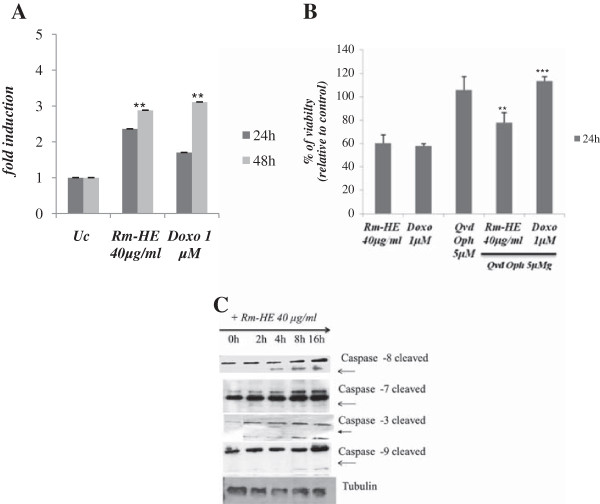
**Effects of Rm-HE on apoptosis induction and activation of caspases in Jurkat cells. (A)** Effect of *Retama monosperma* Hexanic Extract on caspase activity induction on Jurkat cells. Jurkat cells were treated with Rm-HE (40 μg/ml) or 1 μM Doxorubicin (positive control) for 24 h and 48 h and caspase activity was measured as indicated in Materials and Methods. Results indicate the average fold increase ± S.E.M in caspase activity relative to untreated cells from three independent determinations performed in duplicate. **(B)** Effect of *Retama monosperma* Hexanic Extract on viability in the presence of a caspase inhibitor Jurkat cells were pre-incubated for 1 h with 5 μM Q-VD-OPh and Rm-HE (40 μg/ml) was added for 24 h. Doxorubicin was used as positive control. Cell viability is represented as a percentage relative to untreated cells, and data is means ± S.E.M. from three independent determinations performed in duplicate. **(C)** Effect of *Retama monosperma* Hexanic Extract on the expression and cleavage of caspases 8, 7, 3 and 9 in Jurkat cells. 4 × 10cells were treated with Rm-HE (40 μg/ml) for 0, 2, 4, 8 and 16 h. Total and cleaved caspase levels in cellular extracts were detected by immunoblot with specific antibodies. Tubulin was used as an internal control.

Activation of aspartate-specific cysteine proteases also known as caspases is a crucial biochemical event during apoptosis [18,19]. Different caspases are activated during the initiation and execution phases of apoptosis. To investigate if Rm-HE-induced apoptosis is caspase dependent, we performed caspase 3/7 activity assays upon treatment of Jurkat cells with 40 μg/ml Rm-HE for 24 and 48 h. Doxorubicin (1 μM), a conventional drug inducing caspase-dependent apoptosis was used as positive control. Figure 3A showed that both treatments similarly increased caspase activity up to 3-fold upon 48 h. Accordingly, the presence of a specific caspase inhibitor (QVD-OPh) significantly reduced the cytotoxic effects of both Doxorubicin and Rm-HE on cell viability (Figure 3B).

Different caspases are activated by proteolytic cleavage at the initiation and execution phases of apoptosis [20]. To determine the effect of Rm-HE on the activation of caspases, we monitored cleavage of caspases 8, 7, 3 and 9 in Jurkat cells following Rm-HE treatment at different time-points. Results of western blot analysis shown in Figure 3C indicate that Rm-HE treatment caused a strong activation of caspase 8 (detected at 4 h) together with the activation of caspases 7, 3 and 9 in a time-dependent manner. This observation confirms that Rm-HE-induced apoptosis is caspase-dependent and suggests that it operates via the extrinsic pathway.

### Characterizion of the effects of Rm-HE on apoptotic signaling mediators

**Figure 4:**
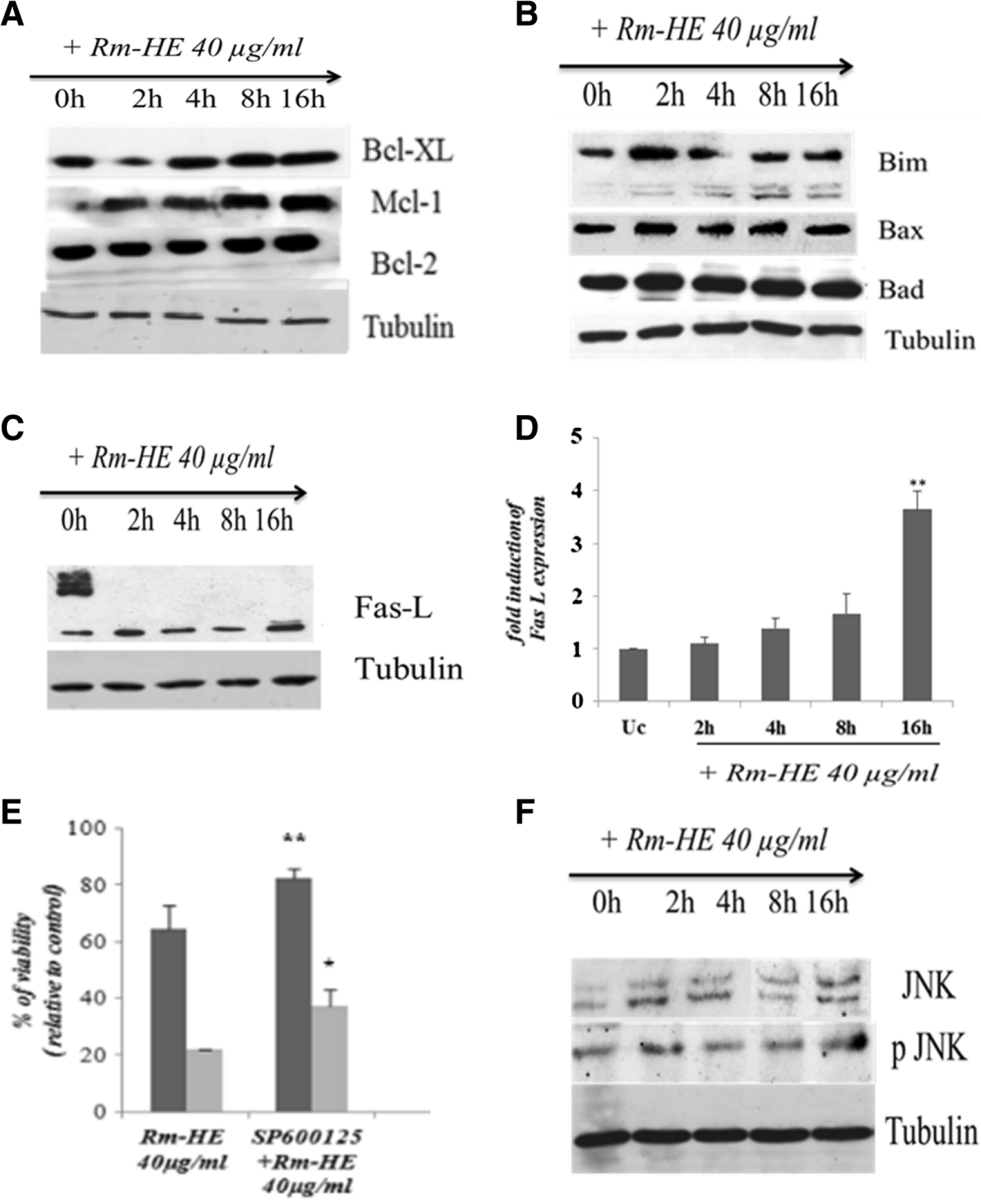
**Characterizion of the effects of Rm-HE on apoptotic signaling mediators. (A)** Effect of Rm-HE on the expression and cleavage of apoptosis-related proteins in Jurkat cells. Expression of the anti-apoptotic proteins Bcl-XL, Mcl- 1 and Bcl-2 was analyzed in extracts of Jurkat cells treated as indicated by immunoblot with specific antibodies. Tubulin was used as an internal control. **(B)** Expression of pro-apoptotic proteins Bim, Bax and Bad was analyzed in extracts of Jurkat cells treated as indicated by immunoblot with specific antibodies. Tubulin was used as an internal control. **(C)** Fas Ligand induction was analyzed in extracts of Jurkat cells treated as indicated by immunoblot with specific antibodies. Tubulin was used as an internal control. **(D)** Statistical analysis for Fas-L expression blots in Rm-HE treated cells. Results indicate the average fold increase ± S.E.M in Fas-L expression relative to untreated cells from three independent experiments. The difference between untreated cells and cells treated for 16 h with Rm-HE are statistically significant (Student’s *t*-test: **P < 0.01). **(E)** Effect of Rm-HE on Jurkat cell viability in the presence of a JNK inhibitor. Jurkat cells were pre-incubated for 1 h with 10μMSP600125 and then 40 μg/ml of Rm-HE were added for 24 h and 48 h, as indicated. Cell viability is represented as a percentage relative to untreated cells. Data is means ± S.E.M. from three independent determinations performed in duplicate. **(F)** Effect of Rm-HE on the JNK phosphorylation in Jurkat cells. Expression of JNK and phospho-JNK were analyzed in extracts of Jurkat cells treated as indicated by immunoblot with specific antibodies. Tubulin was used as an internal control.

Two major pathways are known to mediate cellular apoptosis, death receptor-dependent (extrinsic) and mitochondrial (intrinsic) pathways. Since our results suggest that apoptosis induced by Rm-HE operates via the extrinsic pathway, we further investigated the effects of Rm-HE on the expression of specific proteins involved in either of these pathways. To this end, we analyzed the expression levels of both pro- and anti-apoptotic members of the Bcl-2 family. Western blot analysis indicated that the expression levels of the anti-apoptotic proteins Bcl-xL, Mcl-1, and Bcl-2 [20,21], were not significantly altered upon Rm-HE treatment (Figure 4A). Regarding the pro-apoptotic members of the family, neither Bax nor Bad showed a significant increase upon Rm-HE, whereas Bim levels were modestly increased (Figure 4B). Since caspase-8 is generally activated following assembly of death-inducing signaling complex (DISC) during receptor-mediated apoptosis, we next investigated the expression of Fas ligand, a major inducer of the extrinsic pathway. Figure 4C revealed enhanced Fas-L expression upon exposure to Rm-HE; the statistical analysis of Fas-L blots in Figure 4D showed an expression increase up to 3-fold upon 16 h of treatment. Accordingly, the inhibition of JNK, a key regulator of Fas-L expression, partially reduced the cytotoxic effects of Rm-HE on Jurkat cells (Figure 4E), in agreement with the observed increase in JNK phosphorylation upon time (Figure 4F). Altogether, these observations suggest that apoptosis induced by Rm-HE is activated, at least partially, via the caspase-8-dependent extrinsic pathway.

### Characterization of chemical compounds in *Retama monosperma hexanic* extract

**Figure 5:**
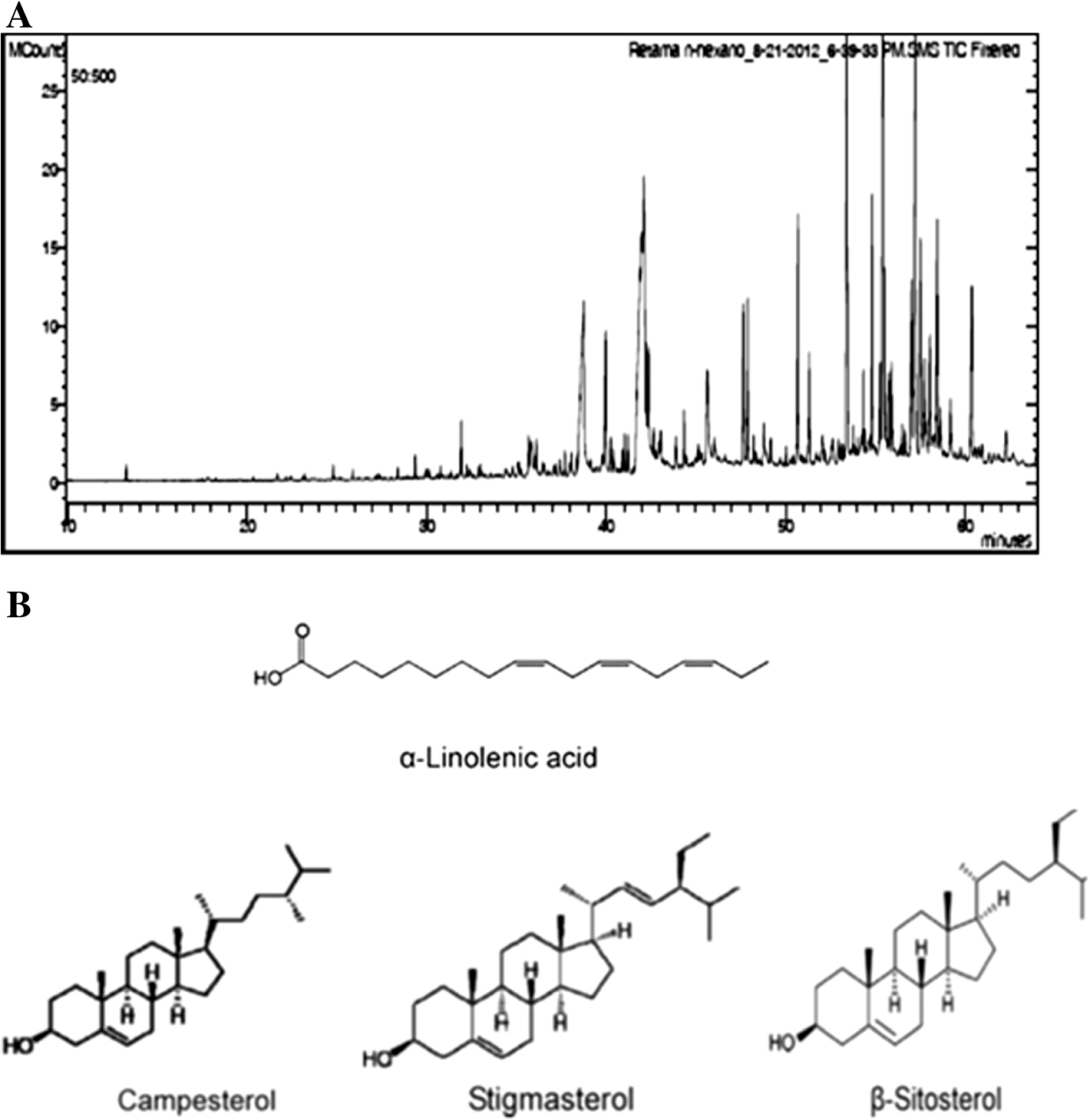
**Characterization of chemical compounds in *Retama monosperma *hexanic extract. A)** Chromatogram of *Retama monosperma* Hexanic Extract (Rm-HE) obtained by CG. Compounds were identified by computer searches in the reference libraries of NIST and Wiley7, and fragmentation patterns were compared with literature data. Rm-HE constituent are shown in Table1. **(B)** Chemical structures of the major bioactive compounds of*Retama monosperma* Hexanic Extract.

In order to identify putative bioactive compounds responsible for these effects, Rm-HE was subjected to Gc-MS analysis (Figure 5). At least 60 compounds were present in Rm-HE, of which 43 were identified using mass spectrometry. Retention time, Cas Numbers and % of identified area are summarized in Table 1. The mass spectra of these compounds were matched with computer searches in the references libraries of NIST08 and Wiley7, and fragmentation patterns were compared with literature and commercially-available products data. The major bioactive compounds identified were α-Linoleic acid (13.97%), Stigmasterol (10.34%), β-Sitosteryl (7.92%) and Campesterol (11.09%). Figure 5B shows their chemical structures.

**Table 1:** Compounds present in hexanic extract of Retama monospermaidentified by CG/MS

**RT**	**Identified compounds**	**cas numbers**	**% Area**
**15,886**	Propanoic Acid,2-[(trimethylsilyl)oxy]-, trimethylsilyl ester	17596-96-2	0,09
**16,386**	HexanoicAcid, trimethylsilylester	14246-15-2	0,04
**16,46**	Acetic acid, (trimethylsiloxy)-, trimethylsilyl ester	33581-77-0	0,027
**21,694**	Silanol,1,1,1-trimethyl-, 1-benzoate	2078-12-8	0,063
**22,167**	OctanoicAcid, trimethylsilylester	55494-06-9	0,063
**24,805**	NonanoicAcidtrimethylsilylester	82326-11-2	0,289
**26,452**	Dicyclohexylamine	101-83-7	0,139
**27,305**	DecanoicAcid, trimethylsilylester	55494-15-0	0,072
**28,004**	malatate	65143-63-7	0,036
**29,279**	TrimethylsilylVanillin	6689-43-6	0,103
**29,368**	(S)-dihydroactinidiolide	17092-92-1	0,257
**31,347**	P-Hydroxybenzoic Acid TMS ether TMS ester	2078-13-9	0,099
**31,927**	DodecanoicAcid, trimethylsilylester	55520-95-1	0,736
**35,036**	Nonanedioic Acid bis(trimethylsilyl) ester	17906-08-0	0,162
**35,441**	SPARTEINE	90-39-1	0,952
**36,12**	TetradecanoicAcidtrimethylsilylester	18603-17-3	0,686
**37,734**	methylpalmitate	112-39-0	0,374
**38,077**	PentadecanoicAcidtrimethylsilylester	74367-22-9	0,519
**39,982**	PalmiticAcidtrimethylsilylester	55520-89-3	2,406
**40,124**	Cytisine/baptitoxine/sophorine/Caulophylline	486-86-2	0,171
**41,748**	HeptadecanoicAcid, trimethylsilylester	55517-58-3	0,259
**42,069**	Aphylline	577-37-7	2,83
**42,221**	Phytol	352332	1,973
**42,996**	LinoleicAcidtrimethylsilyl	56259-07-5	9,988
**43,156**	α Linoleic Acid trimethylsilyl ester	97844-13-8	13,976
**43,52**	OctadecanoicAcid, trimethylsilylester	18748-91-9	1,35
**43,723**	Lupanine	550-90-3	0,212
**45,127**	NonadecanoicAcidtrimethylsilylester	74367-35-4	0,55
**45,59**	oleamide/SLEEPAMIDE	301-02-0	1,291
**46,746**	EicosanoicAcid, trimethylsilylester	55530-70-6	2,853
**47,814**	Monolupine /anagyrine	486-89-5	2,275
**48,903**	Hexadecanoic Acid 2,3-bis[(trimethylsilyl)oxy]propyl ester	1188-74-5	2,569
**49,742**	DocosanoicAcidtrimethylsilylester	74367-36-5	2,804
**50,022**	(-)-PTEROCARPIN	524-97-0	0,329
**50,197**	OctadecanedioicAcid di(trimethylsilyl) ester	22396-20-9	0,438
**51,265**	1-TETRACOSANOL	506-51-4	3,65
**51,684**	Stearic Acid 2,3-bis(trimethylsilyloxy)propyl ester	1188-75-6	2,041
**53,094**	Brucine	357-57-3	0,654
**53,702**	PentacosanoicAcid	506-38-7	0,487
**53,769**	HexacosanoicAcid	506-46-7	2,781
**57,238**	Stigmasteroltrimethylsilylether	14030-29-6	10,34
**57,609**	β-Sitosteryltrimethylsilylether	2625-46-9	7,92
**58,525**	CAMPESTEROL	474-62-4	11,086

## Discussion

Many current research efforts in the field of cancer therapeutics are focused on the development of highly specific agents able to selectively induce apoptosis in cancer, but not normal, cells. In the last two decades there has been a surge of anti-cancer drugs introduced, of which almost half of them are derived from natural sources [22,23]. In Morocco, traditional medicine is supported by one of the world's oldest pharmacopoeia, with an environmental biodiversity that provides a wide arsenal of plant remedies [24]. Currently, plants of the Retama genus have attracted an increasing interest due to their wide range of pharmacological effects including hypoglycemic and diuretic [4,6], cytotoxic [7,8], antioxidant, antiviral [9], antihypertensive [10], anti-inflammatory [11] and antitumor activities [12]. In correlation with our previous observations indicating an anti-leukemic effect of Rm-HE [25], we confirm and extend herein the anti-leukemic and apoptotic-inducing effects of Rm-HE and indicate its cellular mechanism of action on Jurkat cells. For this purpose, a cytotoxic screening with Rm-HE was performed on a panel of established human cancer cell lines including Glioblastoma Multiforme, Acute T cell Leukemia, Mantle Cell Lymphoma, Colon cancer, Prostate Cancer and Osteosarcoma cells, together with non-tumoral control cell lines. In this context, Rm-HE exhibited a dramatic effect on Jurkat cells, which are usually used as a model of acute T cell leukemia, but was essentially ineffective against the other tested cell lines including a B-cell lymphoma cell line (Jeko-1). Interestingly, Acute T cell leukemia is a hematological malignancy characterized by a deregulated expression of apoptosis-related molecules [26].

Interestingly, flow cytometry analysis confirmed that exposure to Rm-HE strongly promoted cell death as indicated by the dramatic time-dependent increase in the proportion of sub-G1 cells. In parallel with cellular death, we observed that the number of cells in S-phase was also reduced, indicating a block in cell cycle progression. DNA damage is one of the major mechanisms behind anticancer drug-induced cell cycle arrest and apoptosis [27,28]. Under normal conditions, genomic integrity in DNA damaged-cells is usually restored through DNA repair. If DNA repair cannot be achieved properly or cells are overwhelmed by sustained damage, apoptosis ensues in order to remove genetically aberrant cells [29,30]. In agreement with the induction of apoptosis, we observed that Rm-HE treatment elicited DNA damage at 4 h as indicated by the increase in H2A.X phosphorylation, a well-established readout for the presence of double strand breaks [31].

In order to better understand the mechanisms of cellular death in response to Rm-HE, we performed flow cytometry analysis upon Annexin-V/ propidium iodide staining to detect and quantify the amount of cells undergoing apoptosis [32]. In this setting, the number of Jurkat cells entering early apoptosis clearly increased in a time-dependent manner, demonstrating that Rm-HE treatment induces Jurkat cell death through the promotion of apoptosis. Since a fundamental requirement for apoptosis to take place is the activation of caspases [18,19], we determined if the enzymatic activity of caspases 3 and 7 was increased in Jurkat cells upon Rm-HE treatment. As expected, Rm-HE clearly induced caspase activation to a similar extent as the conventional chemotherapeutic agent Doxorubycin, and in correlation with this, the inhibition of caspases resulted in a partial protection against Rm-HE-mediated cytotoxicity.

Two fundamental pathways are responsible for the induction of apoptosis: the mitochondrial or intrinsic pathway and the death receptor or extrinsic pathway [32]. Western blot analysis confirmed that both executioner caspases 3 and 7 were cleaved and thus activated by Rm-HE treatment, in parallel with the results obtained in caspase activity assays. Remarkably, Rm-HE treatment rapidly led to procaspase 8 cleavage, which is indicative of activation of the extrinsic apoptotic pathway, an event that was followed by subsequent activation of caspase 9.

In order to understand the involvement of both intrinsic and extrinsic apoptotic pathways in this process, we further investigated molecular events related to both apoptotic pathways in Rm-HE-treated Jurkat cells. To this end, we investigated the activation of both anti- and pro-apoptotic members of the Bcl-2 family. The absence of significant alterations in members of this family in Rm-HE-treated cells pointed to the induction of apoptosis predominantly via the receptor-activated extrinsic pathway. Activation of the extrinsic apoptotic pathway is regulated downstream of the activation of death receptors, and involves ligand-induced formation of death-inducing signaling complex (DISC) that recruits and activates pro-caspase 8. Since a major activator of death receptors in human leukemic cells is Fas-ligand (Fas-L) [33], we next investigated whether Rm-HE treatment could induce Fas-L upregulation. Interestingly, we observed a time-dependent increase in Fas-L in Rm-HE-treated Jurkat cells. A key upstream molecular pathway leading to Fas-L expression is the stress-activated JNK pathway [34,35], which has been shown to lead through Fas-L to caspase 8 and caspase 3 activation [36]. Accordingly, JNK inhibition significantly reduced the cytotoxic effects of Rm-HE in Jurkat cells, suggesting that a JNK/Fas-L/caspase 8/caspase 3 pathway is activated following Rm-HE treatment to promote extrinsic pathway-dependent apoptosis in Jurkat cells. We cannot rule out the contribution of the caspase 8-independent, intrinsic apoptotic pathway to Rm-HE-induced apoptosis, as indicated by the detection of caspase 9 cleavage. However, the robust effects on caspase 8, together with our observations on the involvement of JNK and Fas-L suggest that the extrinsic pathway is the predominant apoptotic pathway in our experimental conditions.

We finally performed GC-MS analysis in order to identify putative bioactive compounds responsible for these cellular effects. Four major bioactives compounds were identified: α-Linoleic acid, Campesterol, β- sitosterol and stigmasterol. When combined together, the last three have been shown to exert cytotoxic activity against cancer cells [37]. Remarkably, Stigmasterol showed an inhibition of cell growth that was not dose-responsive (around 40–50% inhibition between 1.6 and 50 μg/ml) in HS578T breast cancer cells [37]. Additionally, ß -sitosterol and phytol, which is also present in Rm-HE, exhibited a clear cytotoxicity against various cancer cell lines including nasopharyngeal epidermoid carcinoma, breast cancer, cervical carcinoma, colon carcinoma and lung adenocarcinoma cells [38]. These observations support the notion that the combination of several bioactive compounds in Rm-HE exerts its anticancer activity, and pave the way for further research efforts aimed at elucidating if any of the identified compounds could be mainly responsible for these effects.

## Conclusions

Our results suggest that bioactive components of Rm-HE act either alone or in combination to promote cellular apoptosis, predominantly acting via the extrinsic apoptosis pathway, with a strong selectivity against human T cell leukemia. The mechanism whereby this extract induces apoptosis is likely to involve a JNK/Fas-L/caspase 8/caspase 3 pathway in the presence of cell cycle arrest and DNA damage induction. Further studies are required to i) understand the molecular basis of its antileukemic selectivity, ii) determine which of the identified bioactive compounds are responsible for these effects and iii) perform preclinical developmental research aimed at ensuring its safety and efficacy as therapeutic agents.

## Competing interest

The authors declare that they have no competing interests.

## Authors’ contributions

LB performed all experiments and participated in the experimental design, analysis of the data and redaction of the manuscript. CAL participated in the experimental design, analysis of the data and preparation of the manuscript. The plants harvest, identification and extraction were done by NM and YB. SA participated in the analysis of the data and the preparation of the manuscript. SFM participated in the experimental design and the analysis of the data. PV directed the research and edited the manuscript. All authors have contributed and approved the manuscript.

## Pre-publication history

The pre-publication history for this paper can be accessed here:

http://www.biomedcentral.com/1472-6882/14/38/prepub
