# Editorial expression of concern: *Retama monosperma n*-hexane extract induces cell cycle arrest and extrinsic pathway-dependent apoptosis in jurkat cells

**DOI:** 10.1186/s12906-022-03771-2

**Published:** 2022-11-05

**Authors:** Lamiae Belayachi, Clara Aceves-Luquero, Nawel Merghoub, Youssef Bakri, Silvia Fernández de Mattos, Saaïd Amzazi, Priam Villalonga

**Affiliations:** 1 Cancer Cell Biology Group, Institut Universitari d’Investigació en Ciències de la Salut (IUNICS), Edifici Cientificotècnic, Ctra Km 7,5, Valldemossa, Illes Balears, Palma de Mallorca, Spain; 2 Departament de Biologia Fonamental, Institut Universitari d’Investigació en Ciències de la Salut (IUNICS), Universitat de les Illes Balears, Edifici Cientificotècnic, Ctra Km 7,5, Valldemossa, Illes Balears, Palma de Mallorca, Spain; 3 Biochemistry Immunology Laboratory, Faculty of Sciences, Mohammed V-Agdal University, Rabat, Morocco

**Correction: BMC Complement Altern Med 14, 38 (2014)**

10.1186/1472-6882-14-38 **; Published: 24 January 2014**

The Editor is issuing an editorial Expression of Concern for this article. Concerns have been raised that Fig. 3 C and Fig. 4 F show splicing of gels which is no longer considered appropriate practice. Readers should consider interpreting the data with caution. Priam Villalonga agrees with this statement. Lamiae Belayachi, Clara Aceves-Luquero, Nawel Merghoub, Youssef Bakri, Silvia Fernández de Mattos and Saaïd Amzazi have not responded to correspondence from the Editor about this statement.

